# Long 3'UTR of Nurr1 mRNAs is targeted by miRNAs in mesencephalic dopamine neurons

**DOI:** 10.1371/journal.pone.0188177

**Published:** 2017-11-16

**Authors:** Luis Alberto Pereira, Roberto Munita, Marcela Paz González, María Estela Andrés

**Affiliations:** Department of Cellular and Molecular Biology, Faculty of Biological Sciences, Pontificia Universidad Católica de Chile, Santiago, Chile; Universitat des Saarlandes, GERMANY

## Abstract

The development of mesencephalic dopamine neurons and their survival later in life requires the continuous presence of the transcription factor Nurr1 (NR4A2). Nurr1 belongs to the nuclear receptors superfamily. However, it is an orphan member that does not require a ligand to regulate the transcription of its target genes. Therefore, controlling the expression of Nurr1 is an important manner to control its function. Several reports have shown that microRNAs (miRNAs) regulate Nurr1 expression. However, Nurr1 has several splicing variants, posing the question what variants are subjected to miRNA regulation. In this work, we identified a long 3'UTR variant of rat Nurr1 mRNA. We used bioinformatics analysis to identify miRNAs with the potential to regulate Nurr1 expression. Reporter assays performed with the luciferase gene fused to the short (658 bp) or long (1,339 bp) 3'UTR of rat Nurr1 mRNAs, showed that miR-93, miR-204 and miR-302d selectively regulate the mRNA with the longest 3'UTR. We found that the longest variant of Nurr1 mRNA expresses in the rat mesencephalon as assessed by PCR. The transfection of rat mesencephalic neurons with mixed miR-93, miR-204 and miR-302d resulted in a significant reduction of Nurr1 protein levels. In conclusion, Nurr1 mRNA variant with the longest 3'UTR undergoes a specific regulation by miRNAs. It is discussed the importance of fine-tuning Nurr1 protein levels in mesencephalic dopamine neurons.

## Introduction

Nurr1 (NR4A2) is a transcription factor that belongs to the nuclear receptor superfamily. However it is an orphan nuclear receptor since transactivates its target genes in a ligand-independent way. Crystal structure of Nurr1 ligand-binding domain showed that bulky amino acids filling the ligand-binding pocket maintain its transcriptionally active conformation [1]. Therefore, regulating the expression is a major form of controlling Nurr1 function. Nurr1 is codified by an Immediate Early Gene (IEG) whose expression is rapidly induced in the central nervous system (CNS) and other tissues by several kinds of damaging and inflammatory stimuli [2–7]. Even though, several tissues maintain basal levels of Nurr1 as some nuclei in the rat brain [6].

The most important function ascribed to Nurr1 is its absolute requirement for the development of dopamine neurons of the ventral tegmental area (VTA) and substantia nigra (SN) in the brain [8–10]. Nurr1 is also required for the survival of these neurons later in life [11,12]. Nurr1 regulates the expression of several genes important for dopamine neurochemical phenotype, such as the dopamine transporter and tyrosine hydroxylase, among others [13]. In addition, Nurr1 regulates genes important for dopamine neurons survival such as Ret, the tyrosine kinase receptor of the glial-derived neurotrophic factor, GDNF [14,15]. Interestingly, next generation RNA sequence analysis of dopamine neurons from adult Nurr1 knockdown mice, revealed that this transcription factor also regulates the expression of several mitochondria genes [12].

The amount of Nurr1 is relevant for the functions that it plays in the cells. For example, newborn Nurr1 heterozygous mice have half of dopamine tissue content in the striatum and mesencephalon compared to wild-type littermates [10,16], indicating that a full gene dosage of Nurr1 is required for establishing dopamine neurochemical phenotype. Furthermore, heterozygous Nurr1 mice are more susceptible to toxins and show earlier decline of dopamine releaseability in aging animals [17,18]. Interestingly, it was shown that different amounts of Nurr1 regulate different sets of genes in neuronal cell lines [19]. Altogether the data indicate that a certain amount of Nurr1 is required for dopamine neurons to survive and Nurr1 transcriptional activity seems to be regulated by its concentration in the cells.

MicroRNAs (miRNAs) are small non-coding RNA molecules that play key roles fine-tuning the expression of target genes. These small RNA molecules exert their regulatory effects on target mRNAs by either inducing mRNAs degradation or inhibiting translation [20,21]. Usually, target sequences recognized by miRNAs are present in the untranslated 3’UTR of target mRNAs [22]. The generation of a mice deficient in Dicer, the cytosolic RNase responsible for cutting the pre-miRNA precursor to generate the mature miRNA, showed that the production of miRNAs is essential for the development of the mesencephalic dopamine neurons [23]. Consequently, some reports have described the regulation of Nurr1 by selected miRNAs [24,25]. Several Nurr1 splice variants with different length of the 3’UTR have been described [26]. Therefore, we hypothesized that Nurr1 mRNA variants with different 3’UTR length could be subjected to specific miRNAs regulation.

In this study, we describe a new variant of rat Nurr1 mRNA with the longest 3’UTR length, which is present in several tissues including brain. The longest 3’UTR variant of Nurr1 mRNA expresses in the mesencephalon during development along with miR-204, miR-302d and miR-93, which specifically regulate this variant of Nurr1. Co-transfection of miR-204, miR-302d and miR-93 in primary culture of mesencephalon dopamine neurons resulted in a significant reduction of Nurr1 protein.

## Material and methods

### Animals

Adult female pregnant and adult male Sprague-Dawley rats were obtained from the animal facility of the Pontificia Universidad Católica de Chile. All procedures were performed in strict accordance with the guidelines and policies established in the Chilean Institutional Bioethical guide (Comisión Nacional de Investigación Científica y Tecnológica, Conicyt) and were approved by the Ethics Committee of the Pontificia Universidad Católica de Chile and the Ethics Board of the Science Council of Chile (FONDECYT).

### Primary culture of rat mesencephalic dopamine neurons and transfection

Primary culture of mesencephalic dopamine neurons was obtained as described previously [27]. Briefly, ventral mesencephalon of E18 rat embryos were dissected in HANK’s medium and cultured in Neurobasal Medium (21103–049, Gibco, USA) supplemented with 2% (v/v) B-27 supplement (17504044, Gibco, USA), 1 mM L-glutamine (25030–081, Gibco, USA) and 100-units/ml penicillin/streptomycin (15140122, Gibco, USA). Cell cultures were treated with Cytosine β-D-arabinofuranoside hydrochloride (ara-C, C6645, Merck, Germany) 1 μM, during 24 hrs.

The mesencephalic dopamine neurons in culture were transfected using Viromer Red Reagent (Lipocalix, Magdeburg, Germany) according to the manufacturer indications. For immunofluorescence experiments, mesencephalic dopamine neurons were seeded in 24-well plates with 2x10^4^ to 4x10^4^ cells per well. Transfections were carried out in DIV1 and cells were incubated for 48 and 72 h after the transfection and then analyzed by indirect immunofluorescence.

### Immunofluorescence

The immunofluorescence and co-localization analysis were performed essentially as we have described [28]. Briefly, fixed and permeabilized cells were incubated with polyclonal anti-Nurr1 (sc 5568M196, Santa Cruz Biotechnology, Santa Cruz, CA, USA) and/or monoclonal anti-GFP (sc 9996, Santa Cruz Biotechnology, Santa Cruz, CA, USA) overnight, both of them diluted at 1: 500. Also, a monoclonal anti-TH (T1299, Sigma-Aldrich) antibody was used diluted 1: 100. After exhaustive washing, the cells were incubated with the following secondary antibodies: donkey anti-rabbit Alexa Fluor 594 (red) (A21207, ThermoFisher Scientific, USA) and donkey anti-mouse Alexa Fluor 488 (green) (A21202, ThermoFisher Scientific, USA). Immunofluorescence was visualized in a microscope Olympus FV-1000 with a QImaging Micropublisher 5.0 camera coupled to QCapture Pro-software (QImaging, Surrey, BC, Canada).

### RNA extraction and cDNA PCR reaction

Adult male rats were decapitated and samples were taken from the brain (VTA, SN and hippocampus) and from heart, spleen and liver. Tissue samples were placed in TRIzol Reagent (15596018, InvitrogenAmbion, Carlsbad, CA, USA) or Lysis/Binding Buffer (AM1560, mirVANA miRNA isolation Kit, Ambion). Total RNA was extracted with TRIzol Reagent and RNA enriched with small RNAs (100 bp or less) was extracted with mirVANA miRNA isolation kit. About 500 ng of RNA were subjected to reverse transcription using MMLV-RT (28025013, Fermentas International Inc., Burlington, ON, CanadaThermoFisher, USA). The cDNA was subjected to PCR using the following primers to amplify target cDNAs: GFP-F: 5’-TTCTTCAAGGACGACGGCAA-3’, GFP-R: 5’- CTCAGGTAGTGGTTGTCGGG-3’, Nurr1 long variant forward: 5’- AAGGGAACAAGCATGTGACTCTAGG-3’ and Nurr1 long variant reverse: 5’-AACAAGGAATGTTGGACGGTGTTAC-3’.

Tissue samples coming from embryos were collected from pregnant Sprague-Dawley rats at 14 days of gestation. Pregnant rats were decapitated with a guillotine. Embryos were removed from the uterus and placed in cold Hank’s medium. All embryos were decapitated with scalpel and placed in cold Hank’s medium. Embryo heads were located in a stereomicroscope so as to have a sagittal view. The mesencephalon was separated and the ventral (vmDN) was separated from the dorsal (dmDN) mesencephalon.

### miRNA expression

To assess miRNA expression, we used the stem-loop RT-PCR method [29] and the PCR reactions were carried out as described. Briefly, about 10 ng of small RNA was subjected to reverse transcription using MMLV-RT, a universal primer: 5’-ATACTCCAGCTGAGTCTCAACTGGTGTCGTGGAGTC-3’ and a specific reverse primer for each target miRNA: miR-93: 5’- ACACTCCAGCTGGGGGAAGTG CTAGCTCAGCAGTAGG-3’, miR-204: 5’-ACACTCCAGCTGGGCCAGTGATGACAA TTGAACG-3’ and miR-302d: 5’-ACACTCCAGCTGGGCATGCAGTGGCACACAAAG-3’. The cDNA was subjected to PCR assay using the following primers to amplify target miRNAs: miR-93 forward: 5’- ACACTCCAGCTGGGGTCATGGGGGCTCCAAAGT-3’, miR-204 forward: 5’- ACACTCCAGCTGGGGCTACAGCCCTTCTTCATGTG-3’, miR-302d forward: 5’-ACACTCCAGCTTCCCGGTGTAGTAGCCATCAAAGTG-3’ and the universal primer (previously used).

### Cell culture, transfection and luciferase assay

HEK293 cell lines were obtained from American Type Culture Collection (ATCC) and cultured in Dulbecco's modified Eagle's medium (DMEM, 12800–017, Gibco, ThermoFisher, USA), supplemented with 10% fetal bovine serum (16000044, FBS, Gibco, ThermoFisher, USA) and maintained at 37°C and 5% CO2, and supplemented with 1% penicillin/streptomycin (15140122, Gibco, ThermoFisher, USA).

pEZX 3’UTR Nurr1 short vector with the sequence of the Nurr1 3’UTR short variant was purchased from Genecopoeia Inc (I-270 Hi-Tech, Maryland, USA). This vector expresses firefly luciferase gene under the control of the SV40 enhancer, and renilla luciferase gene under the control of the CMV promoter, which was used as a transfection efficiency control. The 3’UTR was cloned 3’ downstream of firefly luciferase gene before the polyadenylation signal. pEZX 3’UTR Nurr1 long vector containing the Nurr1 3’UTR long variant was cloned by Epoch Life Science, Inc. The precursor sequences of the miR-145, miR-302d, miR-130a, miR-204, miR-93, miR-17, miR-455, miR-212 and miR-30a were cloned on pEGP-CE vector, acquired from Cell Biolabs Inc (7758 Arjons Drive San Diego, CA 92126 USA). pEGP-CE codifies a fusion protein of GFP-Puromicine.

HEK293T cells were seeded 24 hours prior to transfection in 24-well plate. The cells were transfected with 100 ng of the reporter plasmid (pEZX-3’UTR Nurr1 short or 3’UTR Nurr1 long) and 4-times the equivalent molar amount of expression plasmids or empty vectors for miRNAs (pEGP-Null). Total amount of DNA (400 ng) was kept constant by adding pBluescript SR (Stratagene). Cells were harvested 48 hours after transfection. Firefly Luciferase activities were normalized to the activity of the internal control Renilla Luciferase. Each set of experiments was performed in triplicate and repeated at least three times. Transfections were carried out using Lipofectamine2000 reagent (52887, Invitrogen, ThermoFisher, USA).

### Calculation of corrected total cell fluorescence (CTCF)

To determine the level of Nurr1 fluorescence in the nuclei of transfected cells, we used the corrected total cell fluorescence (CTCF) calculation [30,31]. Nurr1-like immunofluorescence images were analyzed with ImageJ software. Briefly, for each image the area of the nucleus was selected with drawing/selection tools and the following parameters: Area, Integrated Density and Mean Gray Value were calculated by the program. In the same image a region next to the cell (that has no fluoresce) was selected for background quantification. The calculation of the CTCF was carried out with the following formula: CTCF = Integrated Density–(Area of selected cell * Mean fluorescence of background reading). To ensure that we were exclusively measuring transfected cells we choose only GFP positive cells. The values of CTCF were normalized to each control transfection.

### Identification of target miRNAs

To identify miRNAs candidate to regulate the short variant of Nurr1, we performed two bioinformatics searches, 1) analyses of the expression profiles for miRNAs and Nurr1, to find miRNAs that were not expressed in tissues where Nurr1 is expressed, and vice versa; and 2) using databases, to find miRNA targeting Nurr1 mRNA. The expression profiles used [32, 33] were normalized and analyzed with limma [34, 35] or Affy [36, 37] bioinformatics libraries for R program. Databases used were: TargetScan [38–41], PicTar [42] and MicroCosm [43–45]. The expression profiles gave 120 candidate miRNAs and the database gave 76 candidate miRNAs. Of the 120 miRNAs coming from expression profiles, only 11 coincided with the 76 candidates miRNAs coming from the databases. Of the 11 candidate miRNAs, we choose, according to scores, miR-145, miR-302d and miR-103a for further experimentation.

To identify candidate miRNAs that could regulate the long variant of Nurr1, we used only one parameter: predicted miRNA through databases. The databases used were: TargetScan [38–41], miRDB [46, 47] and microRNA.org [48]. To perform this search, we used the portion of the 3’UTR located downstream of the 3’UTR of the short variant of Nurr1. TargetScan gave 28 miRNAs, miRDB gave 50 miRNAs and microRNA.org gave 69 miRNAs. The intersection of these 3 databases gave 14 miRNAs in common and we choose miR-204, miR-30a, miR-302d, miR-212, miR-93, miR-17 and miR-455 for further experimentation.

## Results and discussion

Increasing evidence support that Nurr1 expression is regulated by miRNAs. In Table 1, we summarized all publications that have described miRNAs that regulate Nurr1. Most of these data comes from stem cells induced to differentiate to neurons. For instance, it was reported that the treatment of human embryonic stem (ES) cells with activing A plus basic fibroblast growth factor (bFGF) induced the repression of Nurr1 along with the induction of several miRNAS, of which miR-302d, miR-217, miR-19a and miR-372, specifically repressed a luciferase reporter gene fused to the 3’UTR of human Nurr1 [24]. However, none of the studies cited in Table 1 addressed the question whether the regulation by miRNAs is restricted to certain Nurr1 splice variants. Several years ago, it was shown that rat and human dopamine neurons produce multiple splice variants of Nurr1 that constitute 20–35% of NR4A2 transcripts, differing mainly in their 5' and 3 'UTRs [26]. Little is known about the function of these Nurr1 variants, besides some of them may play a role as dominant-negative [49] and decrease Nurr1 transactivation by competing for the binding site in the DNA [26].

**Table 1:** Reported miRNAs regulating Nurr1.

Publication	miRNAs	Specie	Tissue	Binding sites	Mutation target site	Verification system
[64]	miR-206	Human	Astrocyte	3'UTR: nucleotide 901.	No	Luciferase assay; Protein levels; Down-regulation of miR-206
[65]	let-7c	Human	Kidney	3'UTR*	No	Luciferase Assay
[66]	miR-409-3p	Human	CRC	3'UTR: nucleotide 900	No	Luciferase Assay
[67]	miR-519d	Human	Trophoblast	3'UTR: nucleotide 1064	Yes	Luciferase Assay
[24]	miR-302d, miR-372, miR-19a miR-217	Human	Human embryonic stem cells	3'UTR: miR-302d and miR-372 nucleotide 1063 miR-19a nucleotide 1044, miR-217 nucleotide 567	No	Luciferase Assay
[25]	miR-132	Mouse	Mouse stem cells	8 exon of Nurr1 cDNA	Yes	Luciferase Assay; Protein levels
[68]	miR-34a/b/c	Human	Human CRC RKO human Colon Carcinoma	3’UTR: nucleotide 1153	Yes	Luciferase Assay; Protein Level

The information about miRNAs binding sites was obtained in the cited publications or searching the databases cited in the publications, except for let-7c*, whose binding site was not reported and could not be found. The verification system used in the cited publications was always a reporter assay with luciferase coupled to the 3’UTR of Nurr1, with the exception of Yang et al. 2012 [19] that used a site located in the 8 exon of Nurr1 cDNA and Bai et al. 2015 [66], which only used the binding site located in the 3’UTR. Protein levels were determined by immunoblotting of Nurr1 in the presence of overexpressed amounts of the miRNA under study.

Most of binding sites for miRNAs described in Nurr1 mRNA (Table 1) are located in the further 3'end of the longest 3’UTR of mice or human Nurr1 mRNAs with the exception of miR-132 [25] that binds in the codifying sequence, and miR-217 [24] that binds around the nucleotide 567 of the longest 3’UTR of human Nurr1 mRNA. These data open the question whether these or other miRNAs control specifically variants of Nurr1 mRNA, with different 3’UTR length.

### Rat Nurr1 has multiple mRNA variants with different 3’UTR length

**Fig 1:**
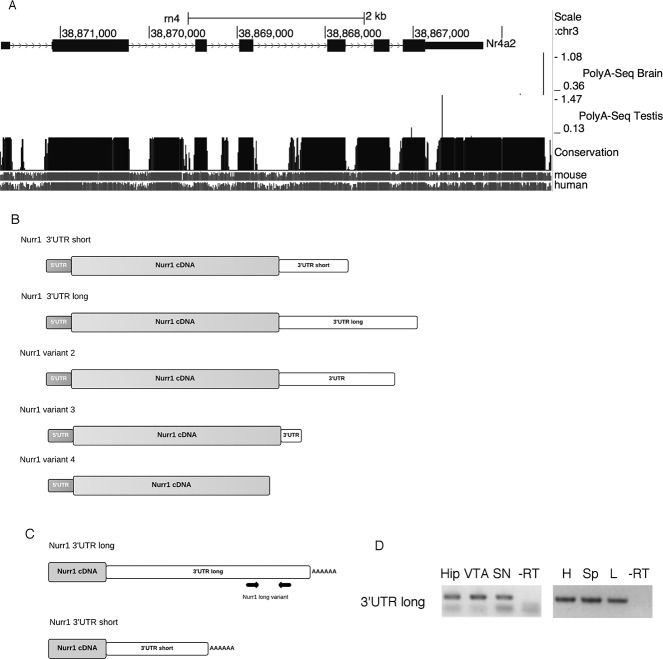
Nurr1 has multiple mRNA variants with different 3’UTR length. **A.** Image from UCSC genome browser depicting the localization of Nurr1 (NR4A2) gene (first panel) in the rat genome (chromosome 3:38865326–38871768). Thick boxes correspond to exons, lines to introns and thin boxes to the 5’UTR on the left end, and to the 3’UTR on right end. The second panel shows PolyA-seq results obtained from brain and testis. The position of the lines indicates the end of the mRNA, and the height of the line indicates the relative abundance. The third panel shows the conservation of the NR4A2 gene sequence of rat compared to human and mouse genomes. **B**. Schematic representation of Nurr1 mRNA variants found in polyA-seq data. Aside from the rat Nurr1 variant already described in the databases, we identified 4 additional mRNA variants. The variant described in the databases was called Nurr1 3’UTR short (UTR length 658 bp), the variant highly expressed in brain was called Nurr1 3’UTR long (3’UTR length 1339 bp). The novel variant found in brain with lower expression was called Nurr1 variant 2 (3’UTR length 1084 bp). The variant highly expressed in testis was named Nurr1 variant 3 (3’UTR length 195) and the variant with the lower expression in testis that lacks 3’UTR and has a shorter seventh exon was named Nurr1 variant 4. **C**. The position of primer pairs to amplify the longest Nurr1 mRNA variant. **D.** RT-PCR products showing the expression of Nurr1 3’UTR long variant. The tissues studied were Hip: hippocampus; VTA: ventral tegmental area; SN: substantia nigra; H: heart; Sp: spleen and L: lung.

The longest rat Nurr1 mRNA described so far possess a 3’UTR of 658 bp, which is half of the longest 3’UTR of human and mouse Nurr1 mRNAs of 1338 bp and 1326 bp, respectively. To look for Nurr1 mRNAs with longer 3’UTR from rat genome, we searched the UCSC genome browser. Fig 1A shows the genome browser interface displaying NR4A2 gene (that codify for Nurr1 protein) localization in the rat genome. Data from poly-A PolyA-Seq experiments conducted by Merck Research Laboratories [50], show that there are four mRNA variants according to the 3’UTR length (distinguished by vertical peaks, Fig 1A). The high similarity of rat Nurr1 3’UTR mRNA with human and mice sequences indicated by peaks of conservation (Fig 1A, lower panel) suggests an important regulatory function for Nurr1 3’UTR.

An mRNA with longer 3’UTR have more chances of having a miRNA seed site and therefore may be subjected to a differential regulation compared with a shorter 3’UTR. Differential targeting of mRNAs with different 3’UTR isoforms was first demonstrated in activated T cells and cancer cells, both of which display global 3ʹUTR shortening compared with non-activated T cells and non-transformed cancer cells, respectively [51,52]. As with miRNA target sites, inclusion or exclusion of mRNA destabilization elements, which often function through RNA-binding proteins, can affect the mRNA stability. Well-characterized motifs include AU-rich elements, GU-rich elements and PUF protein-binding elements [53]

To study the expression of Nurr1 mRNA variant harboring the longest 3’UTR in different tissues, reverse transcriptase PCR assays using a pair of primers that recognizes specifically the distal part of the Nurr1 3’UTR longest variant (Fig 1C and 1D) were carried out. As shown in Fig 1D, Nurr1 3’UTR long is expressed ubiquitously (Hippocampus, VTA, SN, Heart, Spleen and Lung). Albeit, several lines of evidence show that mRNAs with longer 3’UTR are more abundant in the CNS compared to other tissues [54–57], our results show that in the case of Nurr1, the long variant is similarly expressed both in the brain and outside the CNS of the rat. This variant of rat Nurr1 mRNA is probably produced by a phenomenon called alternative polyadenylation (APA). We found various APA sites (APASdb; [58], one located where the rat Nurr1 mRNA polyadenylation site (PAS) was already described and one downstream the described PAS. The PAS already described is referred as first or proximal PAS and the downstream PAS is referred as distal PAS [59]. The proximal and distal PAS for Nurr1 rat mRNA have the canonical sequence AATAAA. The transcripts in the nervous system are characterized by preferential usage of distal PAS [60–63]. The lengthening of 3’UTR trough APA events is more prevalent in mouse and human brain [55].

**Fig 2:**
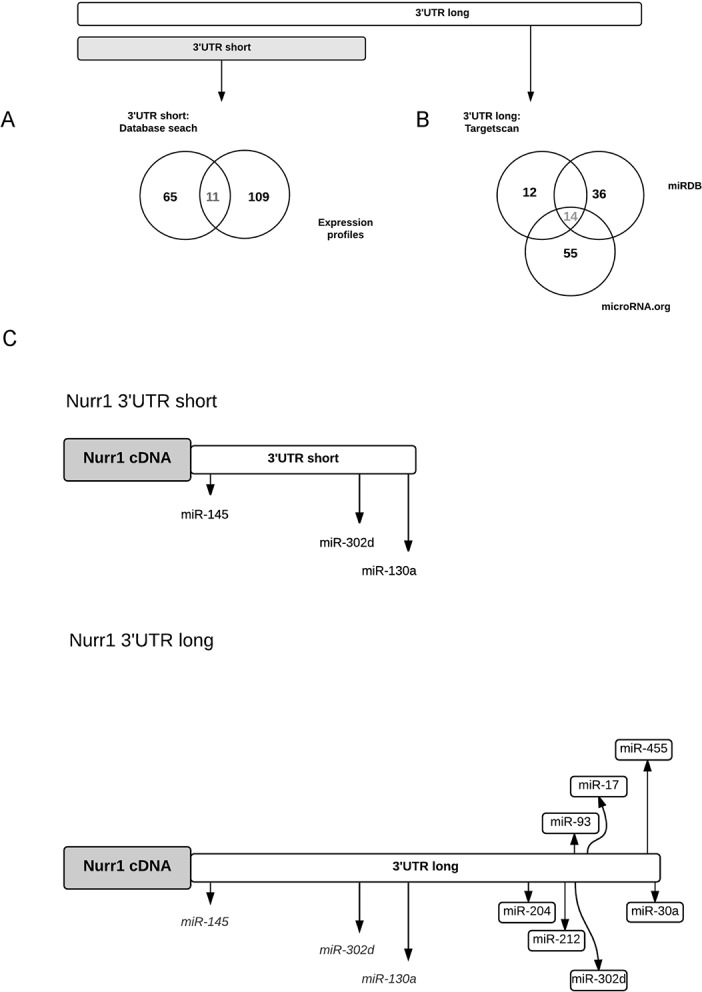
Bioinformatics search for miRNAs candidates to regulate the short or long Nurr1 variant. **A.** Representation of the bioinformatics search for candidate miRNAs targeting Nurr1 short variant as a Venn diagram. The circles represent the parameters of the search: expression profiles and databases search. Gray numbers represent the interactions that were considered for the selection of miRNA candidates. **B**. Representation of the bioinformatics search for candidate miRNAs targeting Nurr1 long variant as a Venn diagram. For this search we only used only database search for candidate miRNAs. The circles represent tree different databases for miRNAs search: Targetscan, miRDB and microRNA.org. The coincidence of the three circles was considered for the selection of miRNAs. **C**. Schematic representation of Nurr1 3’UTR and miRNA seed region localization. The length of Nurr1 3’UTR long is 1339 bp and includes the Nurr1 3‘UTR short (658 bp), shown as white in the image, meanwhile the rest of the 3’UTR is shown in gray. The seed sites of miRNAs selected for the short 3’UTR are: miR-145 in nucleotide 25, miR-302d in nucleotide 465, and miR-130a in nucleotide 606. The seed sites of miRNAs selected for the specific part of the long 3’UTR are: miR-204 in nucleotide 870, miR-212 in nucleotide 1014, miR-93 and miR-302d in nucleotide 1063, miR-17 in nucleotide 1070, miR-455 in nucleotide 1177 and miR-30a in nucleotide 1279.

The fact that Nurr1 mRNA has variants with multiples 3’UTR opened the possibility of differential regulation by miRNAs binding specifically to some of the 3’UTR. A search to look for potential miRNAs that could regulate the expression of Nurr1 3’UTR short and long, or exclusively the long variant was performed. The Venn diagram in Fig 2A and 2B illustrate the criteria used to search and select for potential miRNAs. Two parameters were considered to find miRNAs targeting the proximal common part of Nurr1 3’UTR (short 658 bp): 1) miRNAs predicted by databases and 2) opposites expression profiles between Nurr1 mRNA and miRNAs. The intersection of these criteria resulted in 11 miRNAs (Fig 2A, grey numbers), from which we selected three with higher scores and non-redundant family: miR-145, miR-130a and miR-302d (Fig 2B). To search miRNAs targeting exclusively the Nurr1 3’UTR long (last 600 bp), three different databases to predict miRNAs were interrogated. This search showed 14 common miRNAs among the three databases (Fig 2B, grey number). Seven miRNAs were selected: miR-17, miR-204, miR-455, miR-30a, miR-212, miR-302d and miR-93. Fig 2C shows the localization of the seed sites of these miRNAs on the 3’UTR of Nurr1.

### Nurr1 3’UTR long variant is differentially regulated by miRNAs

**Fig 3:**
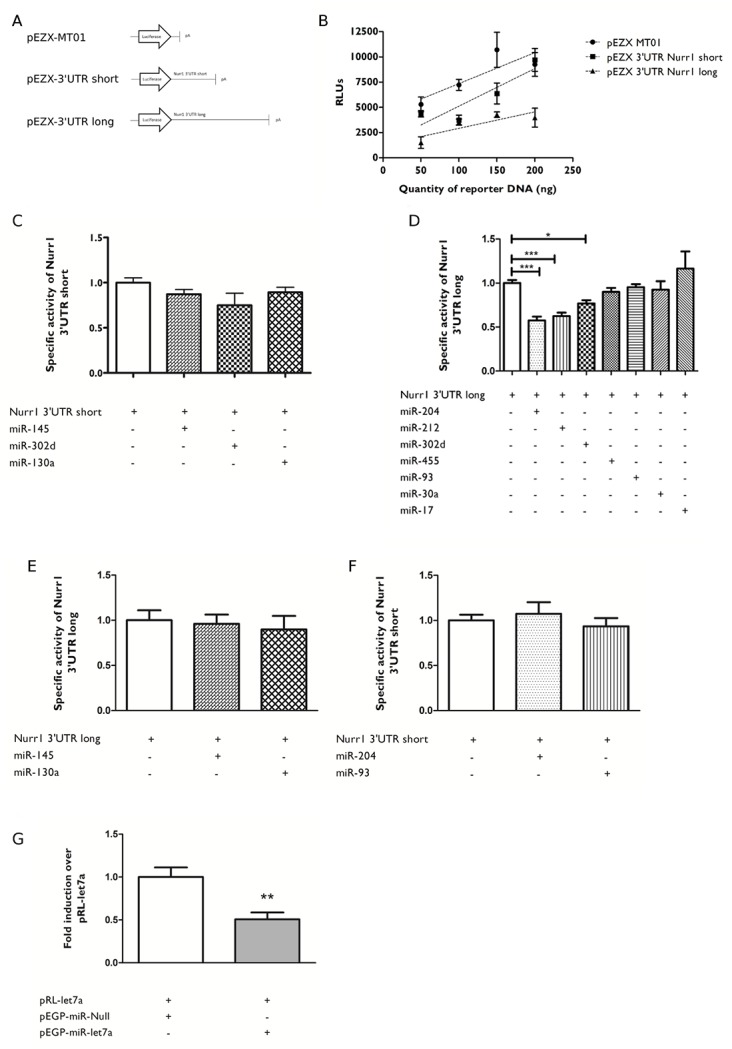
Nurr1 3’UTR long variant is differentially regulated by miRNAs. **A**. Schematic representation of reporter vectors used in the reporter gene assay. pEZX-MT01 is the control vector that codifies the reporter gene luciferase, pEZX-3’UTR short codifies luciferase fused to the Nurr1 3’UTR short and pEZX-3’UTR long codifies luciferase fused to Nurr1 3’UTR long. **B**. Dose/response curve of luciferase activity for pEZX-MT01 (control), pEZX-Nurr1-3’UTR short and pEZX-Nurr1-3’UTR long. **C**. Effect of miR-145, miR-302d or miR-130a overexpression on Nurr1 3’UTR short. **D**. Effect of miR-204, miR-93, miR-17, miR-302d, miR-455, miR-212 or miR-30a overexpression on Nurr1 3’ UTR long. **E**. Effect of miR-145or miR-130a overexpression on Nurr1 3’UTR long. **F**. Effect of miR-204 or miR-93 overexpression on Nurr1 3’UTR short. **G**. pR-let7 is a luciferase reporter vector with two sites for let-7a miRNA localized at the 3'UTR. The graph shows the effect of let-7a miRNA overexpression on pRL-let7a positive control vector. **B, C, D, E, F**. Forty eight hours after transfection the cells were harvested and luciferase activity and amount of proteins were measured. **C, D, E, F**. The results correspond to the average ± SEM of at least 3 independent experiments, performed each in triplicate. The nonparametric Mann-Whitney test was applied to determine statistical significance of the differences. *p<0.05 (Control versus specific miRNA); ***p<0.0001 (Control versus specific miRNA).

To test whether selected miRNAs regulate Nurr1 expression, reporter assays were carried out with the luciferase gene fused to the short or long Nurr1 3’UTR (Fig 3A). A dose-response curve (Fig 3B) shows that luciferase activity is lower for reporters fused to 3’UTR Nurr1 mRNAs compared with the control vector (pEZX-MT01), with the lowest activity for the reporter fused to the longest Nurr1 3’UTR mRNA variant (Fig 3B). These results indicate that the addition of Nurr1 mRNA 3’UTR produces a decrease in the stability of the reporter, suggesting that 3’UTR variants encode seed spots for miRNAs regulation, with a stronger effect for those sites encoded in the specific portion of the longest Nurr1 mRNA 3’UTR variant.

To test the effect of the selected miRNAs, we used luciferase reporters fused to either the short or long 3’UTR of Nurr1 mRNA variants. The results showed that none of the miRNAs selected for the 3’UTR short variant changed significantly luciferase expression (Fig 3C). Conversely, miR-204, miR-93 and miR-302d transfected along with the reporter fused to the long 3’UTR Nurr1 mRNA variant, significantly decreased luciferase expression (Fig 3D). To test the specificity of the effect of the miRNAs, we reversed the assay and tested the miRNAs that recognize specifically the long variant with the reporter fused to the short 3’UTR Nurr1 mRNA variant. As shown in Fig 3F, miR-204 and miR-93 did not change luciferase activity indicating the specificity of their effect controlling the expression of Nurr1 mRNA long variant. Also the miR-145 and miR-130a that did not show the effect on the reporter fused to the short variant also did not modify luciferase activity of the reporter fused to the long variant, indicating that under these conditions, these miRNAs do not regulate Nurr1 expression. To control for the efficiency of our assay, we tested a reporter that possess two seed sites for let7a (pRL-Let7a). As shown in Fig 3G, the co-transfection of pRL-Let7a reporter with the expression vector (pEGP-miR-let7a) significantly reduced luciferase activity demonstrating the efficiency of the reporter system used. Taken together these results indicate that the Nurr1 3’UTR long mRNA variant is specifically regulated by the miR-204, miR-93 and miR-302d. It is surprising that miR-302d did not reduce the expression of the reporter fused to the short variant that has a seed site for this miRNA, but does reduce the expression of the reporter fused to the long variant. It is tempting to suggest that this miRNA requires both seed sites or, there would be a steric hindrance that would preclude miR-302d binding to the seed site located in the short variant.

### miR-204, miR-93 and miR-302d decrease Nurr1 protein levels in cultured dopaminergic neurons

**Fig 4:**
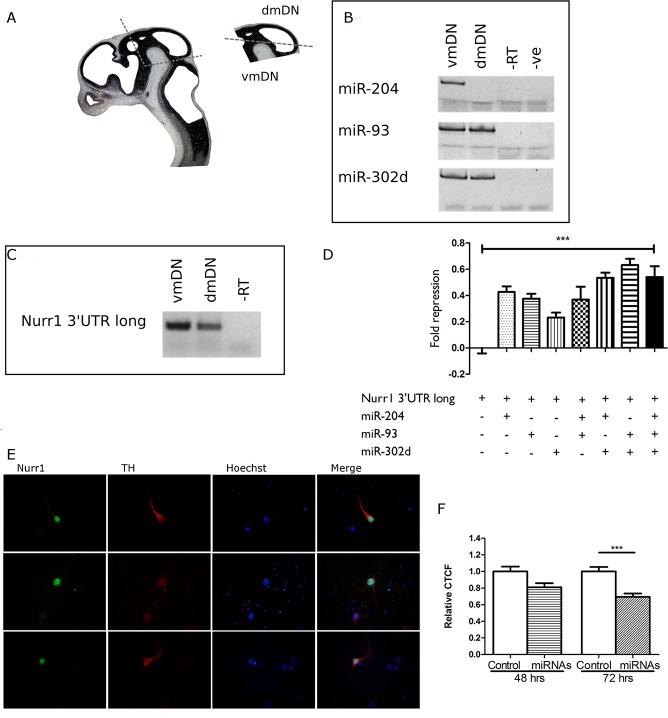
miR-93, miR-204 and miR-302d decrease Nurr1 protein in dopamine neurons. **A**. Sagittal slice of embryonic rat brain at E14 depicting the dissection of the mesencephalon and the separation of ventral (vmDN) and dorsal mesencephalon (dmDN). **B**. Stem-loop RT-PCR showing the expression of miR-93, miR-204 and miR-302d in the developing mesencephalon. **C**. RT-PCR showing the expression of the long 3’UTR variant of Nurr1 mRNA in the developing mesencephalon **D**. Effect of different combination of miR-145, miR-302d and miR-130a on luciferase reporter fused to the long 3’UTR variant of Nurr1 mRNA. **E**. Primary culture of mesencephalic neurons from E18 rat embryo. The cells were cultured until DIV9 and examined by indirect immunofluorescence. TH was detected using a mouse monoclonal antibody (red) and Nurr1 with a rabbit polyclonal antibody (green). Nuclei were marked with Hoechst dye. **F.** Primary culture cells of E18 were transfected with control vector (Control) or an equivalent molar amount of miR-93, miR-204 and miR-302d (miRNAs) at DIV1 and were analyzed at DIV3 (48 hrs after transfection) or DIV4 (72 hrs after transfection) by indirect immunofluorescence. Nurr1 signal intensity was quantified in the nucleus of GFP positive cells. The graph shows the corrected total cell fluorescence (CTCF) of Nurr1 signal in the presence of miRNAs (miRNAs) or control conditions (Control). The nonparametric Mann-Whitney t-test was applied to determine statistical significance of the differences. ***p<0.0001 (Control versus miRNAs at 72 hrs after transfection).

The above results prompted us to study a potential role of miR-204, miR-93 and miR-302d regulating Nurr1 expression in dopamine neurons. Our first approach was to test the hypothesis that in the ventral mesencephalon, which gives origin to dopamine neurons, these miRNAs would not express. To test this hypothesis, we compared the expression of the miRNAs and Nurr1 long 3’UTR mRNA variant between the ventral (vmDN) and dorsal (dmDN) developing mesencephalon (Fig 4A). As shown in Fig 4C, Nurr1 long 3’UTR mRNA variant is expressed in vmDN as well as in dmDN. Unexpectedly, miR-93 miR-204 and miR-302d, which target Nurr1 long 3’ UTR mRNA variant, are expressed in the vmDNA mesencephalon while miR-93 and miR-302d, but not miR-204 express in dmDNA (Fig 4B). These data show that the long Nurr1 mRNA variant coexists with the miRNAs that control its expression, suggesting a complex regulation.

To reveal whether these miRNAs regulate Nurr1 expression in dopamine neurons, we developed a primary culture from embryonic E18 rat mesencephalic cells [27], enriched in tyrosine hydroxylase (TH) positive neurons (Fig 4E). First, it was compared whether the effect of the miRNAs over Nurr1 3'UTR was stronger mixing or keeping them alone. As shown in Fig 4D, combined miRNAs are significantly more efficient decreasing the luciferase reporter fused to the long 3'UTR variant of Nurr1 mRNA. Therefore, to test the effect in a gain of function approach, a mix of the 3 miRNAs (miR-204, miR-93 and miR-302d) were transfected to cultured dopamine neurons. The cultures were transfected in DIV 1 allowing cells to grow for 48 or 72 hours. Nurr1 and GFP proteins expression was analyzed by immunofluorescence and the intensity of Nurr1 signal was quantified in transfected cells (GFP positive cells) by CTCF calculation [30, 31]. The co-transfection of the mix of miRNAs decreased Nurr1 protein levels by 19% at 48 hours, and a stronger significant decrease of 31% was observed at 72 hours, indicating that these miRNAs are effective regulating Nurr1 expression in dopamine neurons.

In conclusion, Nurr1 mRNA with the longest 3'UTR undergoes a specific regulation by miR-204, miR-93 and miR-302d. We propose that the coexistence of long and short 3'UTR variants allows miRNAs to fine tune protein levels of Nurr1. This fine tune of Nurr1 protein levels could be important to regulate the expression of specific set of genes. Indeed, several years ago, it was shown that different set of genes are regulated by Nurr1 in a concentration-dependent manner [19]. Appropriate levels of Nurr1 are also required for the development and survival of dopamine neurons throughout adult life [8–12]. It has been shown that the decay of dopaminergic neurochemical markers as TH, during aging, is associated with down-regulation of Nurr1 expression [69]. Certain diseases correlate with the early loss of Nurr1 in dopamine neurons as it has been observed in brain of addicts [70]. Whether miRNAs are part of the mechanisms provoking Nurr1 drop in these cases deserve further investigation.
